# First-passage-based boundary estimation in functional data

**DOI:** 10.1371/journal.pone.0348779

**Published:** 2026-05-14

**Authors:** Ahmet Tugay Kuzu, Nuri Celik

**Affiliations:** Department of Mathematics, Gebze Technical University, Kocaeli‌‌, Turkey; Menzies School of Health Research: Charles Darwin University, AUSTRALIA

## Abstract

We study a functional threshold-crossing setting in which a population-level boundary evolves smoothly over time and crossing events are observed through subject-specific functional trajectories. Motivated by biomedical and environmental applications where brief excursions above a critical level may trigger risk activation, we formulate boundary recovery as a first-passage identification problem and propose a practical estimation strategy based on smoothing observed crossing pairs. The boundary is represented using penalized cubic B-splines with roughness control, and tuning parameters are selected by generalized cross-validation. We further emphasize the distinction between (i) a time-varying benchmark used to define first-passage times and (ii) a reconstructed conditional crossing-level curve summarizing typical values at the moment of transition. A Monte Carlo study demonstrates that the proposed estimator accurately recovers a variety of smooth threshold shapes under different sample sizes and noise levels, with improved performance as crossing information increases. An illustrative application to country-level age-standardized colon cancer incidence trajectories demonstrates how the framework yields stable empirical boundary reconstructions for male and female series, while explicitly accounting for left-censoring induced by the limited observation window.

## 1 Introduction

Modern biomedical research increasingly relies on high-resolution longitudinal data to examine how disease risk evolves over time. Variables such as environmental exposure, physical activity, and immune response markers are now recorded as continuous temporal profiles rather than isolated measurements. This functional view of biomedical data provides a more nuanced understanding of biological processes while requiring statistical methods that can accommodate complex, time-dependent structures. In chronic diseases such as colorectal cancer, risk may not depend solely on long-term averages of exposure but on brief periods when a biomarker or environmental factor surpasses a critical level. Transient inflammatory episodes or sudden increases in pollutant concentration, for instance, may serve as triggers for disease onset. Traditional regression techniques, including standard functional regression models, are often unable to capture these short-term, threshold-driven dynamics. A suitable modeling framework must explicitly characterize the timing and mechanism by which a functional covariate crosses a time-varying boundary that signals biological activation.

Threshold regression models have long been applied in biomedical and environmental studies to detect critical points at which physiological or environmental variables begin to influence disease risk. For example, [1] used segmented regression to estimate temperature thresholds associated with mortality, while [2] examined cumulative and threshold effects of multiple air pollutants on mortality outcomes. In cardiovascular research, [3] analyzed the nonlinear relationship between systolic blood pressure and mortality among the elderly using a piecewise Cox regression model, and [4] identified structural thresholds in left-ventricular parameters linked to survival. Beyond these individual examples, [5] proposed a general framework for threshold estimation in dose–response regression, and [6] applied data-driven machine-learning techniques to identify health-relevant heat thresholds. Although such models are interpretable and effective for scalar predictors, they assume a fixed threshold and thus fail to capture how‌‌ critical risk levels may evolve dynamically over time. Importantly, these models define thresholds in the covariate space rather than along a time-indexed functional trajectory.

These classical frameworks perform well in simple or static settings but become inadequate when both exposure patterns and critical thresholds vary over time. In many biomedical contexts, brief surges or transient fluctuations in biomarkers and environmental exposures can carry more prognostic information than their long-term averages. However, traditional threshold models are inherently limited by their assumption of fixed cutoffs, which prevents them from capturing the evolving dynamics of biological processes. This limitation has motivated recent efforts to integrate time-varying covariates and more flexible model structures into threshold-based analysis. Within the survival-analysis literature, such developments include threshold regression models with time-dependent covariates ([7]) and functional Cox-type models that allow smooth evolution of covariate effects over time ([8]). Despite these advances, most existing approaches still rely on parametric or static threshold assumptions, making them less suitable for modern high-resolution biomedical data where both exposure trajectories and risk boundaries evolve continuously. In contrast to functional Cox-type models that estimate time-varying effect functions, our objective is to recover the boundary function itself from observed crossing behavior.

In the field of functional data analysis, regression methods for time-varying covariates have been developed to study relationships between functional predictors and scalar or functional responses [9–11]. The functional linear regression model, formalized by [12], represents a scalar outcome as an integral of a functional predictor weighted by a coefficient function. This framework effectively captures global associations between processes but cannot directly model nonlinear activation patterns such as when a biomarker exceeds a critical level. In many applications, activation-type nonlinearities occur locally in time, which limits the interpretability of purely linear formulations.

Recent studies have aimed to introduce greater flexibility into functional regression by relaxing strict linearity assumptions. Penalized functional regression methods provide stable estimation and inference for generalized functional linear models ([13]), while functional generalized additive models allow smooth data-driven departures from linear effects ([14]). These approaches bring functional regression closer to decision boundary modeling but treat threshold behavior as an implicit nonlinear effect rather than as an explicitly modeled boundary function. Therefore, developing modeling frameworks that accommodate both functional effects and smoothly varying threshold functions remains an important direction for modern biomedical applications.

In recent years, [15] introduced the functional threshold autoregressive (fTAR) model, which extends classical autoregressive ideas to functional time series with regime switching. Their framework captures discrete changes between functional regimes by allowing the dynamics of sequential functional observations to depend on whether the process crosses a fixed or data- driven threshold. This approach provides valuable insights for modeling structural shifts in time dependent functional data.

Building on recent developments in threshold-based functional modeling, the present study adopts a data-driven perspective. We consider a functional regression framework in which the threshold is allowed to vary smoothly over time rather than being treated as a fixed constant. The threshold function is estimated from observed first-passage (crossing) events derived from subject-specific functional trajectories. In practice, this estimation is implemented using a nonparametric smoothing approach applied to the observed crossing pairs, yielding a smooth approximation of the empirical crossing-value relationship. Under standard continuity assumptions, these crossing values locally approximate the underlying threshold function. This structural linkage between first-passage equality and boundary recovery forms the theoretical basis of our estimation strategy and provides an identifiability mechanism for the underlying boundary function under continuity assumptions. Specifically, the proposed framework (i) formalizes threshold recovery as a first-passage boundary identification problem, (ii) provides a smooth nonparametric estimator of the boundary function, and (iii) integrates the estimated boundary into a regression structure defined conditionally on crossing times. This approach provides a flexible framework for examining how risk evolves relative to a time-varying boundary rather than a static cutoff.

The proposed model differs fundamentally from [15] in both purpose and structure. Unlike regime-switching models, our formulation does not assume discrete structural breaks but instead focuses on continuous boundary evolution. While the fTAR model is designed to capture regime transitions in sequential functional observations, our framework seeks to identify and estimate a continuously varying threshold function *Q*(*t*) within a regression context. In our formulation, threshold crossings represent activation events in underlying risk dynamics such as the moment a biomarker exceeds a critical level rather than regime shifts in autoregressive dependence. This distinction places the proposed approach within a new class of functional regression problems that integrate first passage theory with smooth, data-driven threshold estimation for dynamic biomedical processes. This perspective expands the scope of functional regression methodology by shifting attention from effect estimation to boundary recovery in dynamic risk processes.

## 2 Threshold regression models‌‌

Let $Y_{i}\in \mathbb{R}$ denote a scalar response and $Z_{i}\in \mathbb{R}$ a scalar covariate. A classical threshold regression model with a fixed threshold τ can be expressed as


$$Y_{i}=\left\{ \begin{array}{cc} \beta_{0}+\beta_{1}Z_{i}+\varepsilon_{i}, & \text{if }Z_{i}\leq \tau ,\\ \beta_{0}+\beta_{1}Z_{i}+\beta_{2}(Z_{i}-\tau )+\varepsilon_{i}, & \text{if }Z_{i}>\tau . \end{array}\right.$$ (1)

where $\varepsilon_{i}\sim 𝒩(0,\sigma^{2})$ and τ∈ℝ is an unknown threshold parameter. In this formulation, the slope of the regression function before the threshold is $\beta_{1}$, whereas after the threshold it becomes $\beta_{1}+\beta_{2}$. Continuity at $Z_{i}=\tau$ follows from the construction of the model, since the additional term $\beta_{2}(Z_{i}-\tau )$ evaluates to zero at the threshold. An equivalent and commonly used representation is the hinge formulation


$$Y_{i}=\beta_{0}+\beta_{1}Z_{i}+\beta_{2}(Z_{i}-\tau {)}^{+}+\varepsilon_{i},$$ (2)

where $\left(x{)}^{+}=\max(0,x\right)$. In this representation, the slope equals $\beta_{1}$ for $Z_{i}\leq \tau$ and $\beta_{1}+\beta_{2}$ for $Z_{i}>\tau$, and the hinge term vanishes at $Z_{i}=\tau$.

In practice, the threshold τ is typically unknown and must be estimated from the data. A common approach is to consider a grid of candidate values $\left\{\tau_{1},\ldots ,\tau_{K}\right\}$ and estimate the model for each candidate threshold. The optimal threshold is selected by minimizing the residual sum of squares (equivalently, maximizing the Gaussian log-likelihood):


$$\hat{\tau }=\arg\min_{\tau_{k}\in \{\tau_{1},\ldots ,\tau_{K}\}}\sum_{i=1}^{n}{\left(Y_{i}-{\hat{Y}}_{i}(\tau_{k})\right)}^{2}.$$ (3)

Here, ${\hat{Y}}_{i}(\tau_{k})$ denotes the fitted value obtained from the threshold regression model estimated under the candidate threshold $\tau_{k}$, with regression coefficients computed by ordinary least squares for that fixed threshold. Under standard regularity conditions, including independent and identically distributed errors and fixed-design covariates, this grid-search procedure yields consistent estimation of τ (see [16]). Throughout this section, we assume independent errors $\varepsilon_{i}$ and treat the covariates *Z*_*i*_ as fixed.

Beyond the scalar regression setting, threshold concepts are closely related to first-passage-time formulations, in which an event is defined by the time at which an underlying stochastic process exceeds a critical boundary. In such settings, each individual follows a process *X*_*i*_(*t*) evolving over time, and the event time is defined as


$$T_{i}=\inf\{t\geq 0:X_{i}(t)\geq \theta \}.$$ (4)

Classical first-passage models are typically embedded within a full survival likelihood framework that accounts for censoring and distributional assumptions on *X*_*i*_(*t*). In the present study, however, we adopt this first-passage representation primarily as a conceptual mechanism linking functional trajectories and threshold crossings, rather than constructing a full survival model.

While classical threshold regression assumes a fixed boundary, modern applications often require more flexible formulations in which decision boundaries evolve over time. This motivates the functional extension developed in the following sections, where the threshold is allowed to vary smoothly and is estimated from observed crossing behavior.

## 3 Functional representation of the threshold

Let each observational unit i=1,…,n be associated with a real-valued functional covariate $X_{i}(t)\in ℋ=L^{2}[0,T]$, observed over a compact interval [0,*T*]. The threshold function Q(t)∈ℋ is assumed to be an unknown but deterministic population-level boundary defined over [0,*T*]. It is not modeled as a stochastic process nor as a subject-specific random function. Rather, *Q*(*t*) represents a common underlying threshold structure shared across observational units. Randomness in the model arises from the stochastic variability of *t*he trajectories *X*_*i*_(*t*) and the resulting crossing times *T*_*i*_, while *Q*(*t*) itself is treated as a fixed but unknown function. The event time *T*_*i*_ is defined as the first threshold crossing


$$T_{i}=\inf\left\{t\in [0,T]:X_{i}(t)\geq Q(t)\right\}.$$ (5)

Assume that each trajectory *X*_*i*_(*t*) is almost surely continuous on [0,*T*] and that *Q*(*t*) is continuous. Under these conditions, whenever *T*_*i*_ < *T*, *t*he crossing occurs without overshoot and satisfies


$$X_{i}(T_{i})=Q(T_{i}).$$ (6)

In practice, functional trajectories are observed on a discrete time grid and may be subject to measurement noise. Consequently, the empirically observed crossing pair (*T*_*i*_, *X*_*i*_(*T*_*i*_)) may only approximately satisfy this equality. In implementation, the empirical crossing time *T*_*i*_ is obtained via linear interpolation between adjacent grid points surrounding the first exceedance. This reduces discretization bias arising from finite time resolution. We additionally verified that the estimated threshold function remains stable under moderate changes in grid density, indicating robustness to discretization effects. To obtain an estimable representation of the threshold, we approximate *Q*(*t*) using a B-spline basis of order *p* with *K* basis functions defined over [0,*T*]:


$$Q(t)=\sum_{k=1}^{K}\beta_{k}\phi_{k}(t),$$ (7)

where $\beta =(\beta_{1},\ldots ,\beta_{K}{)}^{⊤}\in {\mathbb{R}}^{K}$. The number of basis functions *K* is selected via cross-validation, balancing goodness-of-fit and smoothness.

Given observations $\{T_{i},X_{i}(T_{i}){\}}_{i=1}^{n}$, the least-squares estimator is defined as


$$\hat{\beta }=\arg\min_{\beta }\sum_{i=1}^{n}{\left(X_{i}(T_{i})-\sum_{k=1}^{K}\beta_{k}\phi_{k}(T_{i})\right)}^{2}.$$ (8)

Let $\Phi \in {\mathbb{R}}^{n\times K}$ denote the design matrix with entries $\Phi_{ik}=\phi_{k}(T_{i})$ and define


$$𝐗=(X_{1}(T_{1}),\ldots ,X_{n}(T_{n}){)}^{⊤}.$$ (9)

When $\Phi^{⊤}\Phi$ is well-conditioned, the closed-form solution is


$$\hat{\beta }=(\Phi^{⊤}\Phi {)}^{-1}\Phi^{⊤}𝐗.$$ (10)

To ensure smoothness of the estimated threshold function and improve numerical stability, we introduce a roughness penalty of the form


$$\int_{0}^{T}{\left(Q^{\prime \prime }(t)\right)}^{2}dt,$$ (11)

which, under the spline representation, can be written as


$$\beta^{⊤}S\beta ,$$ (12)

where *S* is a positive semi-definite penalty matrix determined by the chosen basis. The penalized estimator is therefore given by


$${\hat{\beta }}_{\lambda }=\arg\min_{\beta }\left\{\sum_{i=1}^{n}{\left(X_{i}(T_{i})-\sum_{k=1}^{K}\beta_{k}\phi_{k}(T_{i})\right)}^{2}+\lambda \beta^{⊤}S\beta \right\},$$ (13)

where λ>0 is a smoothing parameter selected via generalized cross-validation (GCV) or *K*-fold cross-validation. The corresponding regularized solution is


$${\hat{\beta }}_{\lambda }=(\Phi^{⊤}\Phi +\lambda S{)}^{-1}\Phi^{⊤}𝐗.$$ (14)

As a flexible descriptive alternative, we consider a LOESS estimator applied to the observed crossing pairs (*T*_*i*_, *X*_*i*_(*T*_*i*_)):


$${\hat{Q}}_{\text{LOESS}}(t)=\sum_{i=1}^{n}w_{i}(t)X_{i}(T_{i}),$$ (15)

where the weights *w*_*i*_(*t*) are determined via local linear regression with span selected by cross-validation. Local linear fitting mitigates boundary bias near the edges of [0,*T*].

The LOESS estimator provides a nonparametric estimate of the conditional mean function


$$m(t)=E[X_{i}(T_{i})|T_{i}=t].$$ (16)

Under the structural first-passage formulation and assuming crossings occur without systematic overshoot and with mean-zero variability around the boundary, we have


$$E[X_{i}(T_{i})|T_{i}=t]=Q(t).$$ (17)

Under these assumptions, smoothing the crossing pairs yields a consistent estimator of the underlying deterministic threshold function *Q*(*t*). We emphasize, however, that the pairs (*T*_*i*_, *X*_*i*_(*T*_*i*_)) arise from a selection mechanism induced by the crossing condition; identifiability of *Q*(*t*) therefore relies on the struc*t*ural first-passage assumption and the absence of systematic bias in the crossing mechanism. To further clarify the identifiability of the boundary function *Q*(*t*) under the proposed sampling scheme, we emphasize that *t*he estimation relies on the structural properties of the first-passage framework. Specifically, under the assumption that each trajectory crosses the boundary for the first time at *T*_*i*_, the observed pairs (*T*_*i*_, *X*_*i*_(*T*_*i*_)) provide direct information about the boundary at the crossing time. In this setting, identifiability is supported by the fact that the first-passage mechanism induces a well-defined correspondence between the crossing time and the boundary level, provided that the underlying trajectories evolve continuously and the crossing occurs uniquely. Importantly, while the sampling scheme is inherently selective, it does not introduce systematic bias in the estimation of *Q*(*t*) as long as the crossing mechanism is governed by the same underlying stochastic structure across units. Therefore, the identifiability of *Q*(*t*) is no*t* derived from random sampling of the full trajectories, but rather from the structural constraint imposed by *t*he first-passage condition, which effectively anchors the boundary at observed transition points. This perspective aligns with the objective of reconstructing the boundary function from transition events, rather than performing full distributional inference.

## 4 Simulation study

In this section, we evaluate the finite-sample performance of the proposed functional threshold crossing framework via Monte Carlo simulations. The objective is to assess how accurately the deterministic threshold function *Q*(*t*) can be recovered from observed first-passage times under varying sample sizes, noise levels, and crossing densities.

The simulation design proceeds as follows:

1. For each scenario, a deterministic and smooth threshold function *Q*(*t*) is specified over t∈[0,1]. The explicit functional form is provided within each scenario.
2. For each subject i=1,…,n, a functional trajectory *X*_*i*_(*t*) is generated on a fine equidistant grid over [0,1]. The structural form of *X*_*i*_(*t*) and the noise specification depend on the scenario under consideration.
3. For each generated trajectory, the first-passage time *T*_*i*_ is computed according to the definition given in Section 3. When no crossing occurs within the domain, the observation is recorded as a non-crossing trajectory. Simulation parameters are calibrated such that the proportion of non-crossing trajectories remains below % 7 in all configurations; this proportion is explicitly monitored and reported.
4. The threshold function is estimated using the penalized spline approach described in Section 3, including the same B-spline basis construction, roughness penalty, and smoothing parameter selection via GCV.
5. The procedure is repeated *R* = 1000 times for each combination of sample size n∈{25,50,100,200} and noise level σ∈{0.01,0.05,0.10}.
6. Estimation accuracy is evaluated using the mean squared error (MSE) computed on a fine evaluation grid over [0,1]. We report the average MSE and its standard deviation across replications. The mean squared error (MSE) is computed as

$$MSE=\frac{1}{m}\sum_{j=1}^{m}{\left(\hat{Q}(t_{j})-Q(t_{j})\right)}^{2},$$ (18)

where $\{t_{j}{\}}_{j=1}^{m}$ denotes a fine evaluation grid over [0,1].

### 4.1 Scenario I: Linear functional covariates with quadratic threshold

We consider the time domain t∈[0,1], discretized into 200 equidistant grid points. The true threshold function is defined as


$$q(t)=-4(t-0.5{)}^{2}+0.8,$$

which yields a smooth downward-opening parabola centered at *t*=0.5.

For each replication and each subject i=1,…,n, we generate linear trajectories of the form


$$X_{i}(t)=d_{i}t+\varepsilon_{i}(t),$$

where $d_{i}\sim Uniform(0,1)$ independently across subjects. The noise term is generated as


$$\varepsilon_{i}(t)\sim 𝒩(0,\sigma^{2}),$$

independently across subjects and time points.

For each trajectory, the first-passage time is computed as


$$T_{i}=\inf\{t\in [0,1]:X_{i}(t)\geq q(t)\}.$$

**Fig 1:**
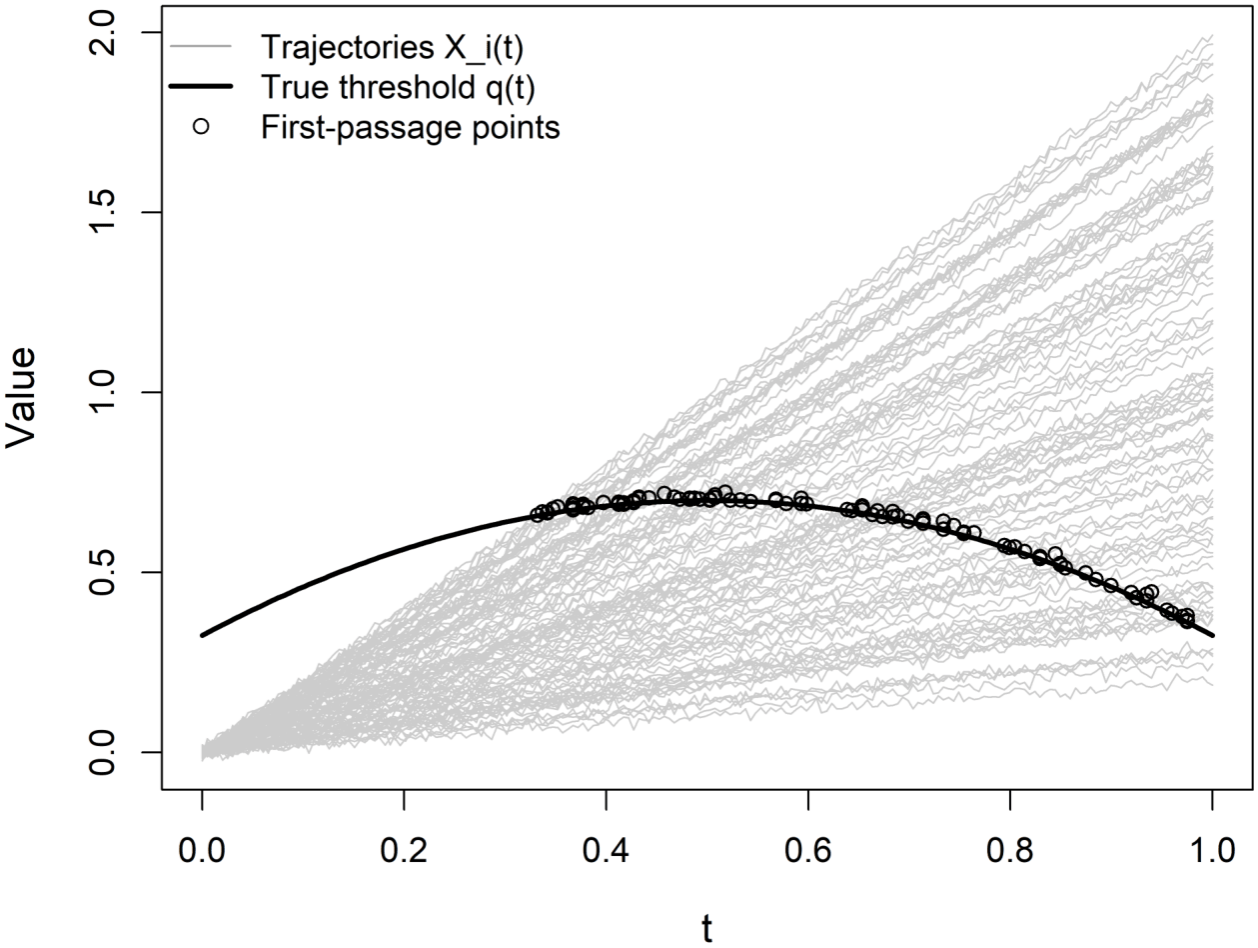
Illustration of the data-generating process in Scenario I.

Parameter values are chosen so that the proportion of non-crossing trajectories remains negligible across all configurations. The threshold function is then estimated using the penalized cubic B-spline approach described in Section 3, with the smoothing parameter selected via generalized cross-validation (GCV). Fig 1 illustrates the data-generating mechanism employed in Scenario I, including the structure of covariate trajectories and the threshold function.

Table 1 presents the average MSE and corresponding standard deviations obtained from 1000 Monte Carlo replications under varying sample sizes n∈{25,50,100,200} and noise levels σ∈{0.01,0.05,0.10}. The censoring proportion in each configuration is also reported. The results show a clear improvement in estimation accuracy as the sample size increases. For each fixed noise level, the MSE decreases with larger *n*, indicating the stabilizing effect of additional crossing information. As expected, higher noise levels lead to increased estimation error, reflecting the reduced signal-to-noise ratio.

**Table 1:** Average MSE and standard deviation under varying sample sizes and noise levels for Scenario I.

Sample Size (*n*)	Noise SD (σ)	Average MSE	MSE Std. Dev.	Censoring Proportion
25	0.01	0.05536	0.09307	0.01
	0.05	0.10759	0.16151	0.01
	0.10	0.26728	0.42528	0.02
50	0.01	0.02043	0.03091	0.02
	0.05	0.03922	0.05790	0.03
	0.10	0.09379	0.13343	0.03
100	0.01	0.00761	0.01163	0.04
	0.05	0.01507	0.02245	0.04
	0.10	0.04046	0.06244	0.07
200	0.01	0.00270	0.00511	0.05
	0.05	0.00296	0.00590	0.05
	0.10	0.01216	0.02236	0.07

**Fig 2:**
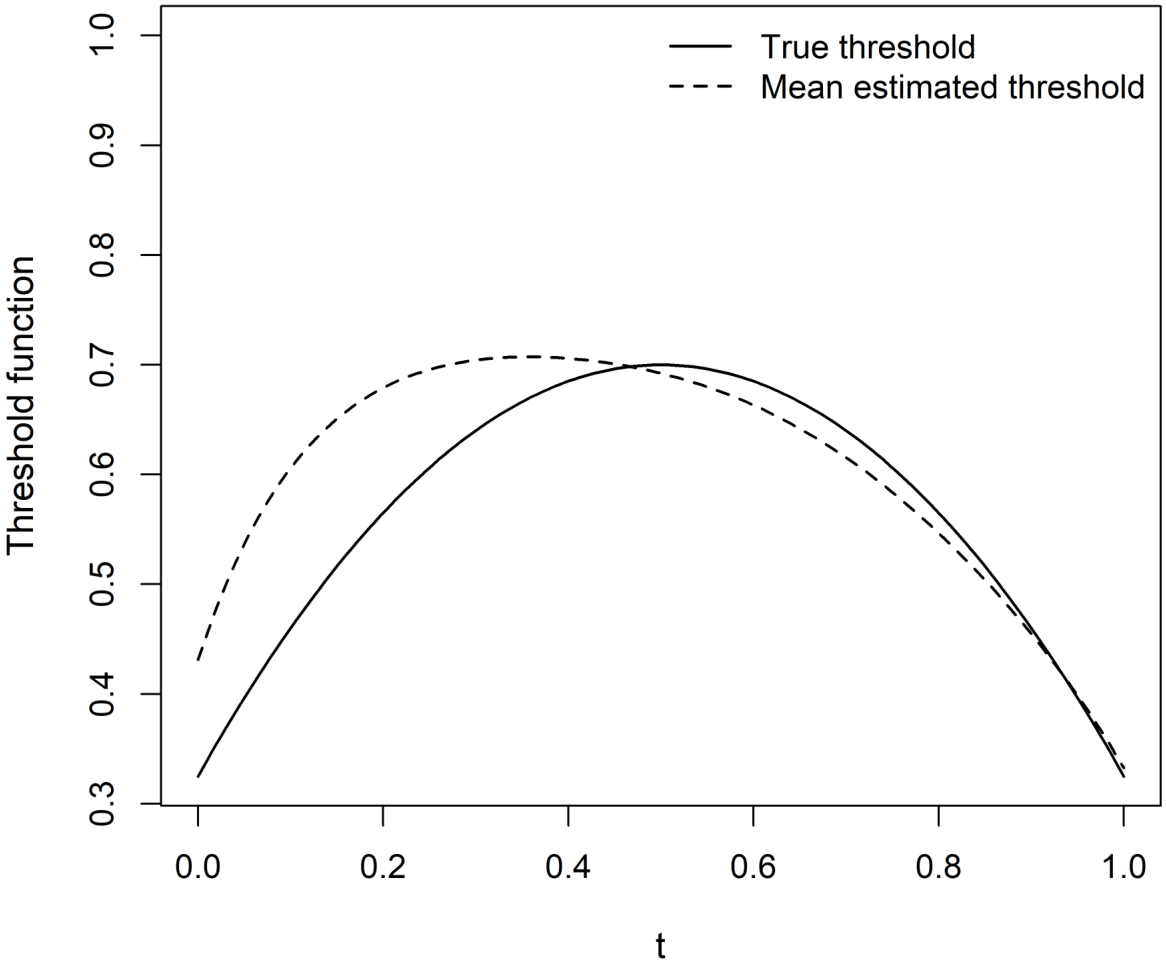
True threshold function and estimated mean threshold for Scenario I.

Fig 2 displays the true threshold function *Q*(*t*), the average estimated threshold $\hat{Q}(t)$, and the pointwise standard deviation band around the estimator with *n* = 100 and σ=0.05. The figure shows that the estimator closely follows the underlying smooth pattern of the true threshold, particularly in the interior region of the domain. Slight deviations are observed near the boundaries, likely due to limited observations close to the edges and the influence of spline boundary effects. Overall, the figure confirms the consistency and stability of the proposed estimation approach in moderate noise and sample size settings.

### 4.2 Scenario II: Nonlinear growth covariates with linear decreasing threshold

**Fig 3:**
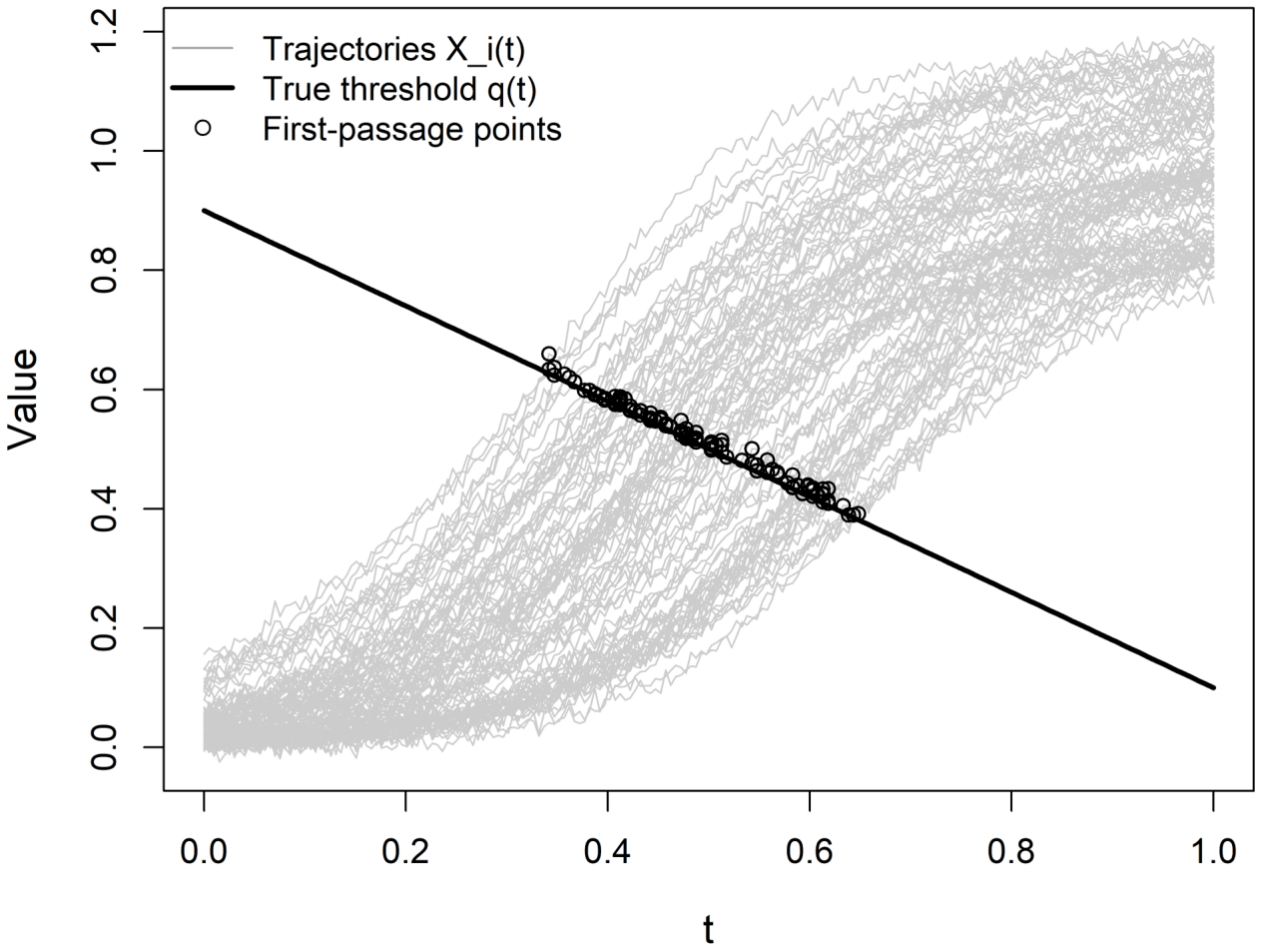
Illustration of the data-generating process in Scenario II.

We again consider t∈[0,1] discretized into 200 equidistant grid points. In this scenario, each trajectory follows a logistic-type growth pattern. For each subject i=1,…,n, we generate


$$X_{i}(t)=\frac{a_{i}}{1+\exp\{-b_{i}(t-c_{i})\}}+\varepsilon_{i}(t),$$

where


$$a_{i}\sim Uniform(0.8,1.2),\;b_{i}\sim Uniform(5,10),\;c_{i}\sim Uniform(0.3,0.7),$$

independently across subjects. The noise process is generated as


$$\varepsilon_{i}(t)\sim 𝒩(0,\sigma^{2}),$$

independently across subjects and time points. The threshold function is specified as a linearly decreasing curve


q(t)=−γt+δ,

with (γ,δ)=(0.8,0.9). Parameter values are chosen such that non-crossing trajectories remain rare across simulation settings. The threshold function is estimated using the same penalized cubic B-spline procedure described in Section 3, with smoothing parameter selected via GCV. Fig 3 provides an illustration of the data-generating mechanism in Scenario II, where each functional covariate *X*_*i*_(*t*) follows a sigmoidal trajectory governed by a logistic growth model, and the threshold function *q*(*t*) decreases linearly over time. The plot demonstrates how the covariate curves evolve and intersec*t* the threshold function, highlighting the dynamic structure of crossing events used for estimation.

Table 2 reports the average MSE and its standard deviation under varying sample sizes and noise levels. The threshold function is estimated using the penalized cubic B-spline procedure described in Section 3. For each fixed noise level, the MSE decreases as *n* increases, reflecting improved estimation accuracy with more observed crossings. For a fixed sample size, larger noise levels lead to higher estimation error, consistent with a reduced signal-to-noise ratio. In all configurations, the censoring proportion equals zero.

**Table 2:** Average MSE and standard deviations for Scenario II.

Sample Size (*n*)	Noise SD (σ)	Average MSE	MSE Std. Dev.
50	0.01	0.00087	0.00127
	0.05	0.00669	0.00925
	0.10	0.01998	0.02942
100	0.01	0.00041	0.00050
	0.05	0.00313	0.00352
	0.10	0.00907	0.01084
200	0.01	0.00025	0.00025
	0.05	0.00201	0.00185
	0.10	0.00542	0.00547

**Fig 4:**
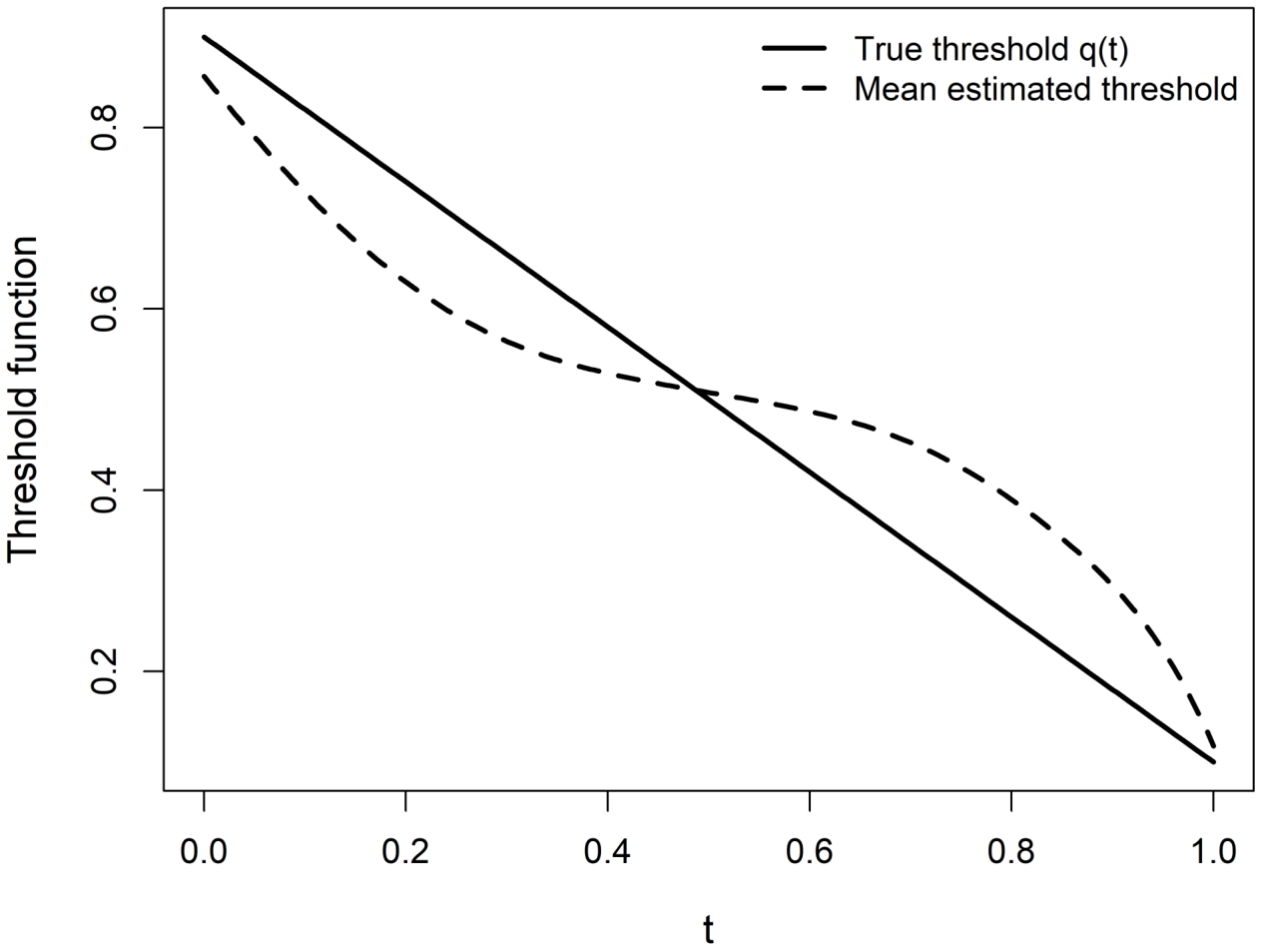
True threshold function and estimated mean threshold for Scenario II.

Fig 4 displays the true threshold for *n* = 100 and σ=0.05.

### 4.3 Scenario III: Parabolic covariates with piecewise-linear threshold

**Fig 5:**
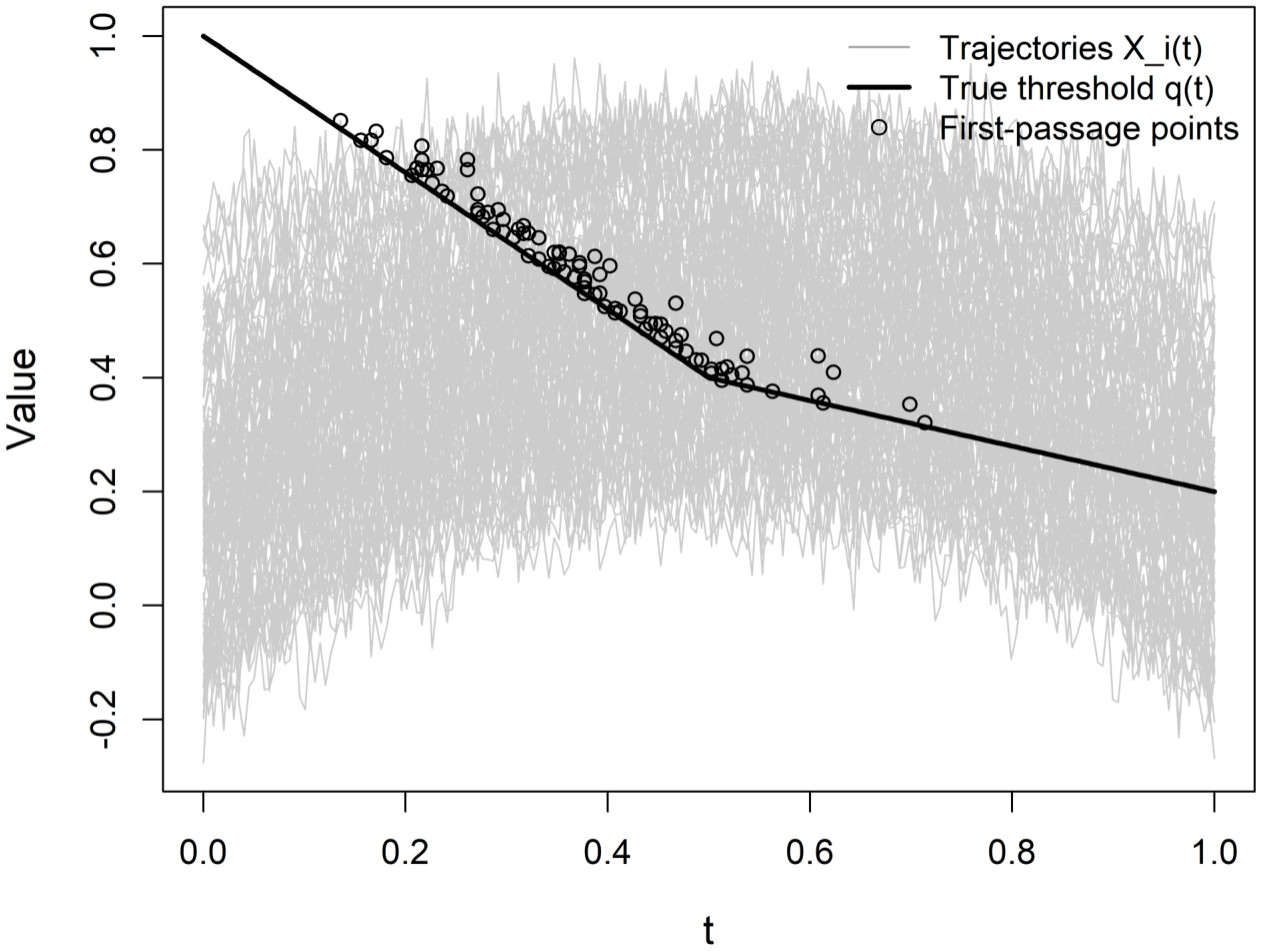
Illustration of the data-generating process in Scenario III.

The threshold function is defined as a continuous piecewise-linear curve with a single change-point at *t*_0_ = 0.5:


$$q(t)=\left\{ \begin{array}{cc} \alpha_{1}+\beta_{1}t, & 0\leq t\leq t_{0},\\ \alpha_{2}+\beta_{2}t, & t_{0}<t\leq 1, \end{array}\right.$$

where $\left(\beta_{1},\beta_{2})=(-1.2,-0.4\right)$. Continuity at *t*_0_ is enforced by setting


$$\alpha_{2}=\alpha_{1}+(\beta_{1}-\beta_{2})t_{0},$$

with $\alpha_{1}=1.0$. This yields a steeper negative slope on [0,*t*_0_] followed by a flatter decline on (*t*_0_,1]. For each subjec*t* i=1,…,n, trajectories are genera*t*ed as


$$X_{i}(t)=a_{i}(t-0.5{)}^{2}+b_{i}+\varepsilon_{i}(t),$$

where


$$a_{i}\sim Uniform(-2,-0.5),\;b_{i}\sim Uniform(0.2,0.8),$$

independently across subjects. The noise process is generated as independently across subjects and time points. Fig 5 provides a schematic view of the data-generating mechanism in Scenario III, where predictor trajectories follow parabolic shapes with additive noise, and the threshold function exhibits a continuous piecewise-linear form with a smooth change in slope around the midpoint.

Table 3 reports the average MSE and its standard deviations. The censoring proportion for each configuration is also provided. As expected, the MSE decreases with increasing sample size, reflecting improved stability in the reconstruction of the threshold function *q*(*t*). For fixed *n*, higher noise levels lead to larger estimation errors, consistent with a lower signal-to-noise ratio.

**Table 3:** Average MSE and standard deviations for Scenario III.

Sample Size (*n*)	Noise SD (σ)	Average MSE	MSE Std. Dev.	Censoring Proportion
25	0.01	0.00025	0.00032	0.01
	0.05	0.00043	0.00053	0.01
	0.10	0.00082	0.00068	0.01
50	0.01	0.00013	0.00018	0.01
	0.05	0.00022	0.00025	0.02
	0.10	0.00043	0.00037	0.02
100	0.01	0.00008	0.00010	0.02
	0.05	0.00014	0.00017	0.02
	0.10	0.00028	0.00024	0.02
200	0.01	0.00006	0.00007	0.02
	0.05	0.00010	0.00011	0.02
	0.10	0.00019	0.00019	0.04

**Fig 6:**
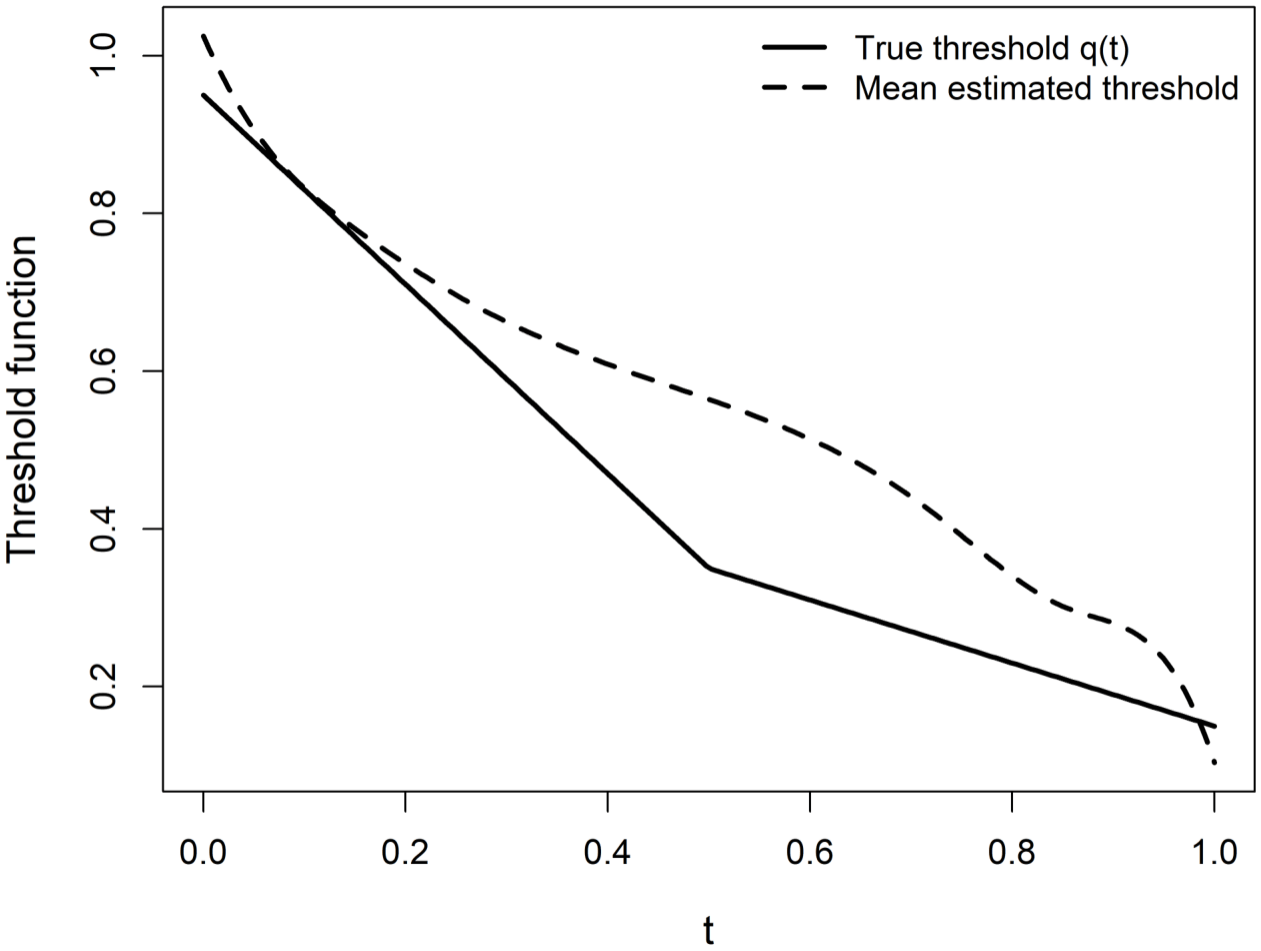
True threshold function and estimated mean threshold for Scenario III.

Fig 6 shows the average estimated threshold curve and its pointwise standard deviations, alongside the true piecewise-linear threshold function.

## 5 Illustrative application: Empirical boundary reconstruction in colon cancer incidence

In this illustrative application, we demonstrate how the proposed crossing-based framework operates on real epidemiological data. The dataset consists of annual age-standardized colon cancer incidence rates (per 100,000 population) observed from 1998 to 2016 across multiple countries. For each country *i*, the incidence trajectory *X*_*i*_(*t*) is treated as a functional observation over calendar time *t*. The data were obtained from the World Heal*t*h Organization (WHO) Cancer Database, which provides country-level age-standardized incidence rates for major cancer types. The sample consists of countries for which complete annual incidence data over the study period were available. Countries with missing observations or inconsistent reporting across years were excluded to ensure comparability of functional trajectories.

Because both the overall level and the cross-country dispersion of incidence rates evolve over time, defining a high-incidence regime using a single constant threshold would be inappropriate. Instead, we construct a time-varying benchmark derived from the empirical cross-sectional distribution of incidence rates. For each calendar year *t*, we compute the cross-sectional *p*-quantile across countries,


$$q_{p}(t)={Quantile}_{p}\{X_{1}(t),\ldots ,X_{n}(t)\},$$

with *p* = 0.65 in our implementation. This produces a year-specific empirical boundary reflecting the upper tail of the distribution in that year.

To reduce year-to-year variability and ensure smooth temporal evolution, the raw quantile curve *q*_*p*_(*t*) is smoothed using a penalized cubic B-spline estimator as described in Section 3. Let ${\hat{q}}_{p}(t)$ denote the resulting smooth benchmark. The smoothing parameter is selected via generalized cross-validation (GCV), and a second-order difference penalty is used to control roughness.

The first-passage time for each country is then defined relative to this smoothed, time-varying benchmark,


$$T_{i}=\inf\{t\in [1998,2016]:X_{i}(t)\geq {\hat{q}}_{p}(t)\}.$$

Countries whose incidence trajectories already exceed the benchmark at the initial observation year, are treated as left-censored and are excluded from the boundary regression illustration below. For the remaining countries, we construct the crossing pairs (*T*_*i*_, *X*_*i*_(*T*_*i*_)), representing the observed incidence level at the time a country enters the upper-incidence regime defined by ${\hat{q}}_{p}(t)$. Based on these pairs, we estimate a smooth function


$$Q^{⋆}(t)=E[X_{i}(T_{i})|T_{i}=t],$$

using a penalized cubic B-spline regression with smoothing parameter selected by GCV. This curve summarizes the typical incidence level observed at the moment of transition as a function of calendar time.

It is important to distinguish between the two curves introduced above. The function ${\hat{q}}_{p}(t)$ defines the time-varying benchmark used to determine first-passage times, whereas $Q^{⋆}(t)$ describes the conditional mean incidence level at the time of crossing. These two objects serve different purposes and need not coincide. The presence of countries whose incidence levels already exceed the benchmark at the beginning of the observation window requires careful interpretation. For such countries, the true first-passage time occurs prior to 1998 and is therefore not observed within the available time horizon. Formally, these cases correspond to left-censored observations with *T*_*i*_ ≤ 1998. Because the objective of this empirical illustration is to reconstruct the boundary function from observed transition events rather than to perform survival or hazard modelling, we restrict estimation of $Q^{⋆}(t)$ to countries for which the first crossing is observed within the study window. To further clarify this point, the exclusion of units with crossings prior to the observation window is a deliberate modeling choice. Restricting the sample to observed crossings ensures that each included observation provides direct information about the boundary at the time of transition. While this reduces the effective sample size, it avoids introducing partially observed events that could bias the estimation of $Q^{⋆}(t)$. Consequently, the reconstructed boundary reflects the conditional mean crossing level among countries whose transition into the upper-incidence regime occurs during 1998–2016. A full survival analysis explicitly incorporating left- or interval-censoring would require additional modelling assumptions and lies beyond the scope of this illustrative application.

**Fig 7:**
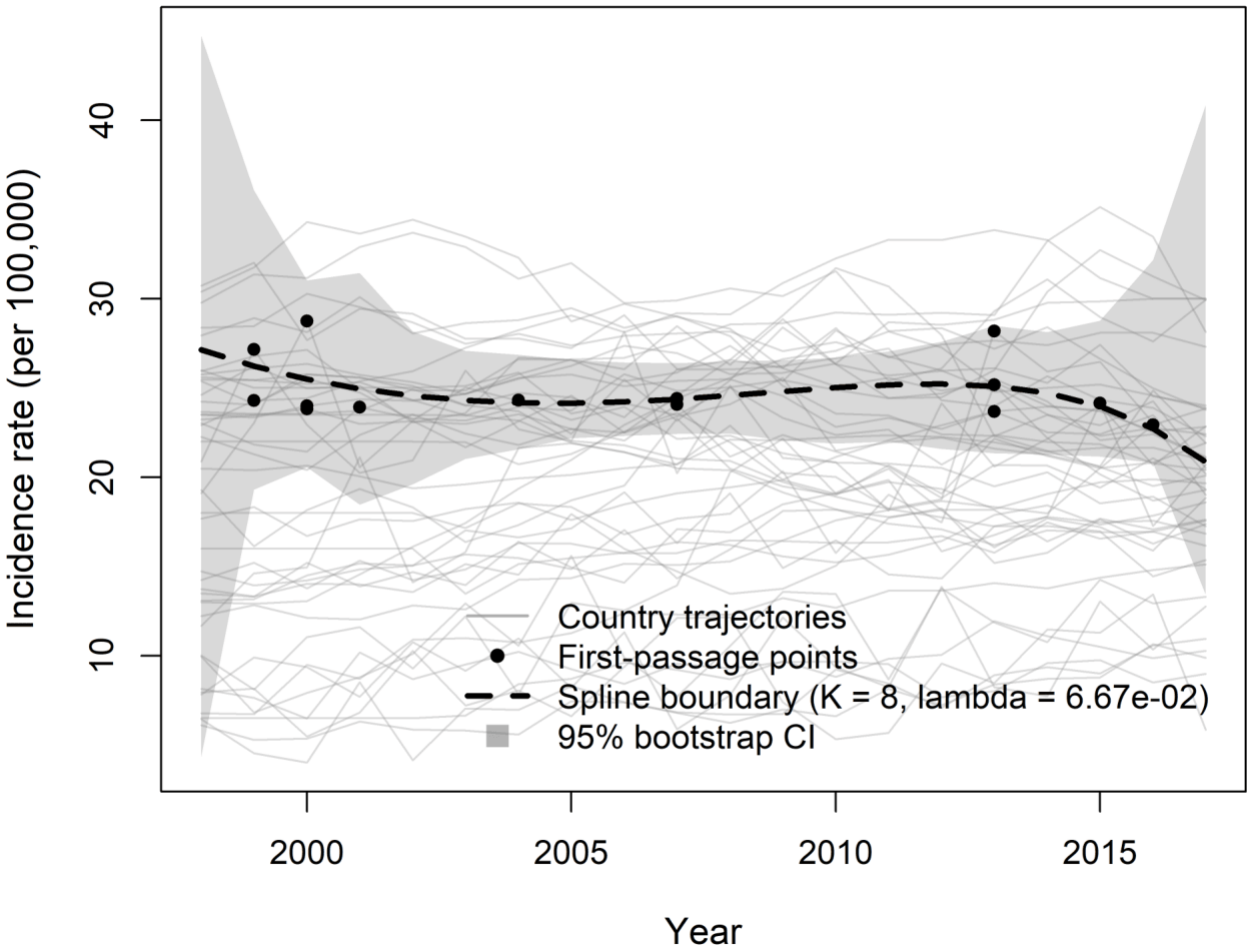
Threshold function first-passage points, and male incidence trajectories.

For the male sample, we set *p* = 0.65 and constructed the year-specific empirical benchmark as the cross-sectional *p*-quantile of incidence rates across countries at each calendar year. The raw quantile curve was smoothed using a penalized cubic B-spline with *K*_*q*_ = 10 basis functions and a second-order difference penalty, with the smoothing parameter selected via generalized cross-validation (GCV). First-passage times were then defined relative to this smoothed time-varying benchmark. Under this definition, 14 countries exhibited an observed first crossing within 1998–2016, while 14 countries were already above the benchmark in 1998 and were therefore treated as left-censored ($T_{i}\leq 1998$) and excluded from the boundary estimation. The boundary curve based on the observed crossing pairs was estimated using a penalized cubic B-spline with *K* = 8 basis functions and GCV-selected smoothing parameter. For males, GCV selected a relatively strong penalty ($\lambda =10^{2}$), producing a stable boundary that appears approximately flat with a mild downward tendency over time (see Fig 7). This pattern indicates that, among countries whose transition into the upper-incidence regime occurs during the observation window, the typical incidence level at entry remains broadly stable across years rather than exhibiting a pronounced upward drift.

**Fig 8:**
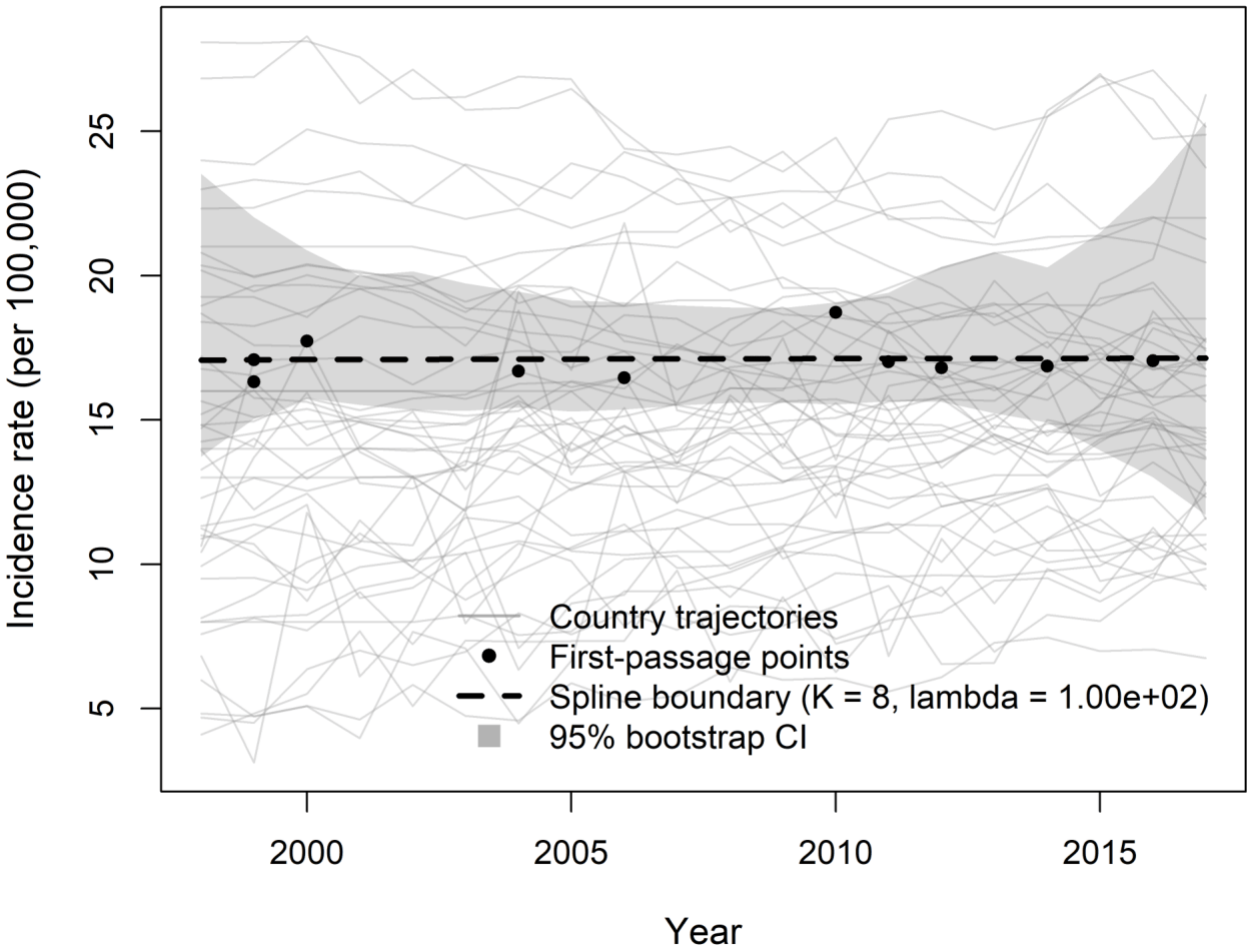
Estimated threshold function for female incidence trajectories, first-passage points, and raw country-specific curves.

For the female sample, the same procedure was applied with *p* = 0.65. The year-specific cross-sectional quantile curve was smoothed using a penalized cubic B-spline with *K*_*q*_ = 10 basis functions and a second-order difference penalty, with the smoothing parameter selected via generalized cross-validation (GCV). First-passage times were defined relative to the resulting smoothed benchmark. In the female sample, 10 countries exhibited an observed first crossing within 1998–2016, while 17 countries were already above the benchmark in 1998 and were therefore treated as left-censored and excluded from the boundary estimation. The boundary curve based on the observed crossing pairs was estimated using a penalized cubic B-spline with *K* = 8 basis functions and GCV-selected smoothing parameter. As in the male sample, GCV selected a strong penalty ($\lambda =10^{2}$), yielding a stable and smooth boundary estimate (see Fig 8). Compared to the male sample, the smaller number of observed transitions and the larger proportion of left-censored countries indicate that, for females, a substantial share of countries had already entered the upper-incidence regime prior to 1998. The reconstructed boundary therefore reflects the conditional entry levels among the subset of countries experiencing transition during the observation window.

To assess sensitivity with respect to the choice of the quantile level, we repeated the analysis using alternative values of *p* in a reasonable neighborhood of the baseline specification (e.g., *p* = 0.60 and *p* = 0.70). The resulting smoothed benchmarks and reconstructed boundary curves exhibited qualitatively similar shapes, and the overall interpretation remained unchanged. While the exact number of observed crossings varied slightly across specifications, the estimated conditional boundary displayed the same broadly stable temporal pattern. These results indicate that the main findings are not driven by the particular choice of *p* = 0.65.

In addition, to assess the uncertainty associated with the estimated boundary functions, 95% bootstrap confidence bands are presented in Figs 7 and 8. These bands are obtained by resampling countries with replacement and reconstructing the boundary function under each bootstrap replicate. As expected, the confidence bands are relatively wider near the beginning and the end of the observation window. This behavior reflects the well-known boundary effect in spline-based estimation, as well as the limited number of observed first-passage events near the temporal edges. Overall, the results indicate that the estimated boundary functions are stable over the central time region, where the data provide the strongest support.

## 6 Conclusion

This paper developed a crossing-based framework for reconstructing a smoothly evolving threshold function from functional trajectories. The central idea is to exploit first-passage behavior: when trajectories are continuous and crossings occur without systematic overshoot, the observed crossing levels provide localized information about the underlying boundary. We operationalized this idea using penalized cubic B-splines, yielding a stable and interpretable estimator with smoothing selected by generalized cross-validation. Our simulation study showed that the proposed procedure can recover a range of boundary shapes in finite samples, with estimation accuracy improving as sample size increases and noise decreases. The results also highlighted the practical role of crossing density: configurations producing more observed crossings lead to tighter reconstructions, whereas sparse crossings naturally limit what can be learned about the boundary in certain regions of the domain. In the colon cancer illustration, we demonstrated a pragmatic approach for defining first-passage events via a time-varying, cross-sectional quantile benchmark and for reconstructing a smooth conditional boundary based on observed transitions. The empirical reconstructions for males and females were stable and exhibited broadly similar temporal patterns. Importantly, the analysis makes explicit that countries already above the benchmark at the start of the observation window represent left-censored cases; the reconstructed boundary should therefore be interpreted as conditional on transitions observed during 1998–2016 rather than as a full event-time model. Extending the framework to a dedicated survival likelihood that jointly models left- and right-censoring, or incorporating external covariates to explain cross-country heterogeneity, are natural directions for future work. Overall, the proposed methodology provides a flexible descriptive tool for boundary recovery in functional settings, complementing existing functional regression approaches by shifting attention from estimating time-varying effects to reconstructing the time-varying boundary that governs activation-type dynamics.
